# Natural Biopolymer-Based Delivery of CRISPR/Cas9 for Cancer Treatment

**DOI:** 10.3390/pharmaceutics16010062

**Published:** 2023-12-30

**Authors:** Meng Lin, Xueyan Wang

**Affiliations:** Department of Pharmacy, West China Hospital, Sichuan University, Chengdu 610041, China

**Keywords:** CRISPR/Cas9, natural biopolymer, gene delivery, tumor therapy

## Abstract

Over the last decade, the clustered, regularly interspaced short palindromic repeats (CRISPR)/CRISPR-associated protein 9 (Cas9) system has become the most promising gene editing tool and is broadly utilized to manipulate the gene for disease treatment, especially for cancer, which involves multiple genetic alterations. Typically, CRISPR/Cas9 machinery is delivered in one of three forms: DNA, mRNA, or ribonucleoprotein. However, the lack of efficient delivery systems for these macromolecules confined the clinical breakthrough of this technique. Therefore, a variety of nanomaterials have been fabricated to improve the stability and delivery efficiency of the CRISPR/Cas9 system. In this context, the natural biopolymer-based carrier is a particularly promising platform for CRISPR/Cas9 delivery due to its great stability, low toxicity, excellent biocompatibility, and biodegradability. Here, we focus on the advances of natural biopolymer-based materials for CRISPR/Cas9 delivery in the cancer field and discuss the challenges for their clinical translation.

## 1. Introduction

Cancer is one of the leading causes of disease-associated mortality, with a rising incidence worldwide [1]. Although many therapeutic methods have been used in cancer treatment, such as chemotherapy, radiotherapy, surgery, and targeted therapy, the overall therapeutic outcome remains unsatisfactory. Therefore, developing new therapeutic means is urgently needed. With advances in our understanding of cancer biology, more and more evidence indicates that genetics play a vital role in cancer pathogenesis and growth through the accumulation of multiple genetic and epigenetic mutations [2,3]. Therefore, correcting the genetic mutation of tumor cells by gene editing technology will revolutionize the field of cancer treatment. Gene therapy can alter the disease-related gene by introducing exogenous nucleic acids for gene deletion, gene replacement, and gene suppression. For example, small interfering RNA (siRNAs) and microRNA (miRNA) suppress the gene expression by triggering the mRNA degradation or affecting the stability of mRNA [4]. However, the gene suppression effect mediated by siRNA or miRNA is transitory and may be associated with the risk of off-target effects. On the contrary, the clustered regularly interspaced short palindromic repeats (CRISPR)/CRISPR-associated protein 9 (Cas9) systems, which render facile and precise gene editing, have been widely utilized to treat genetic diseases, including cancer. The advantage of the CRISPR/Cas9 system over traditional gene therapies is that gene knockin or gene knockout is permanently and precisely executed at the genome level. The natural CRISPR-Cas9 system consists of three components: Cas9, CRISPR RNA (crRNA), and transactivating crRNA (tracrRNA) [5]. The crRNA usually has a 20-nt protospacer sequence for target DNA binding and an extra part used for tracrRNA complementary pairing. The tracrRNA has two functional parts for crRNA and Cas9 protein binding. To facilitate the experimental design, crRNA and tracrRNA have been fused into a single RNA chain—a single guide RNA (sgRNA) for mammalian genome editing with CRISPR/Cas9 [6]. The complex formed by the Cas9 and sgRNA is called ribonucleoprotein (RNP), which is the key to CRISPR/Cas9-mediated genome editing: the Cas9 endonuclease can target the specific sites under the guidance of sgRNA and modify the genome in a site-specific manner. Compared with conventional gene editing tools, such as ZFNs (zinc finger nucleases) and TALENs (transcription activator-like effector nucleases), CRISPR/Cas9-mediated gene editing is more effective, flexible, and precise [2]. Owing to its potential to manipulate the genome, CRISPR/Cas9 has been widely used to manipulate the oncogenes [7,8], the cell death-related genes [9], the immune-related genes [10,11], and the tumor microenvironment-associated genes [12] in cancer treatment both in preclinic and in the clinic. The first successful clinical trial using CRISPR/Cas9 was initiated at West China Hospital, Sichuan University, in 2016. In this trial, the immune checkpoint regulator PD-1 of the T cells was first knocked out ex vivo using the CRISPR/Cas system. Then, the PD-1 knockout T cells were infused back into the patients for non-small cell lung cancer (NSCLC) treatment [13]. Over ten clinical trials are now currently underway [3,14], including CRISPR/Cas9-based therapy for acute lymphoblastic leukemia [15], transthyretin amyloidosis [16], sickle cell disease, and β-thalassemia [17,18], and the initial results have been promising [14].

As mentioned above, the key to CRISPR/Cas9-mediated genome editing is RNP. In practice, the CRISPR/Cas9 system can be introduced in the targeted cells in three forms: DNA, mRNA, and RNP. Each of these delivery forms has advantages and disadvantages in efficiency and accuracy. In the DNA or plasmid-based CRISPR/Cas9 system, the Cas9 and sgRNA cassettes can be packed in one plasmid and express RNPs in the cells after transcription and translation. Thus, plasmid-mediated gene editing requires a relatively long time, and the durable expression of RNPs is associated with a higher risk of off-target effects [19]. An alternative approach is to use Cas9 mRNA and sgRNA. This mRNA-based CRISPR/Cas9 system enables the swifter genome editing, as it bypasses the process of transcription. Besides, the transit expression of RNPs may reduce the risk of off-target mutagenesis. The most straightforward approach is to deliver Cas9/sgRNA ribonucleoprotein complexes, which can directly edit the genome without the need for transcription or translation, thus resulting in the quick onset of genome editing. 

To achieve efficient gene editing, the CRISPR/Cas9 system (DNA, mRNA, or RNP) has to overcome multiple barriers in vivo despite their differences in mode of action. In circulation, they are prone to degradation and clearance when exposed to proteases and nucleases in the plasma and can not maintain their bioactivity before reaching the target sites. Besides, as macromolecules, they can not easily pass through the biological membranes via passive diffusion due to their high molecular weight. The massive negative charge of DNA and mRNA further hinders cellular entry. Inefficient endosomal escape and nuclear entry also restrict the genome editing efficiency mediated by CRISPR/Cas9. Therefore, it is urgent to develop ideal delivery strategies to overcome the multiple barriers mentioned above. Currently, most of these delivery strategies are based on physical approaches (microinjection, electroporation, nucleofection, and membrane deformation) and viral vectors [20]. Despite their high efficiency, the safety and immunogenicity issues remain to be solved. Alternatively, non-viral vectors, especially nanomaterials, have drawn attention due to their biocompatibility, stability, and biodegradability. Various nanomaterials have been utilized in the delivery of CRISPR/Cas9 machinery, including liposomes [21], micelles [22], lipid nanoparticles [23,24], exosomes [25], polymers [26,27] and cell-penetrating peptides (CPP) [28]. Among these nanomaterials, natural biopolymer-based nanocarriers are preferred due to their biocompatibility, biodegradability, and low immunogenicity. Besides, natural biopolymers are abundant in the biomass and are easily accessible from a range of sources, such as plants, animals, and microorganisms. Moreover, they can be further modified to improve their solubility and targeting ability, their ability to condense DNA, and their stability in vivo. 

As many reviews have detailed the progress of nanotechnology in CRISPR/Cas9 delivery [3,29,30], in this review, we do not intend to elaborate on the various nanomaterials for CRISPR/Cas9 delivery. Instead, we will focus on the advances of natural biopolymer-based materials for CRISPR/Cas9 delivery. Firstly, we will introduce the mechanism of CRISPR/Cas9 and its three forms of delivery, with an emphasis on their multiple barriers of delivery. Besides, we will provide an overview of the delivery methods for CRISPR/Cas9, highlighting the natural biopolymer-mediated delivery. Finally, we will discuss the challenges of natural biopolymer-based nanomaterials in gene editing and clinical translation.

## 2. CRISPR/Cas9-Mediated Gene Editing System

### 2.1. Mechanism of CRISPR/Cas9 

**Figure 1:**
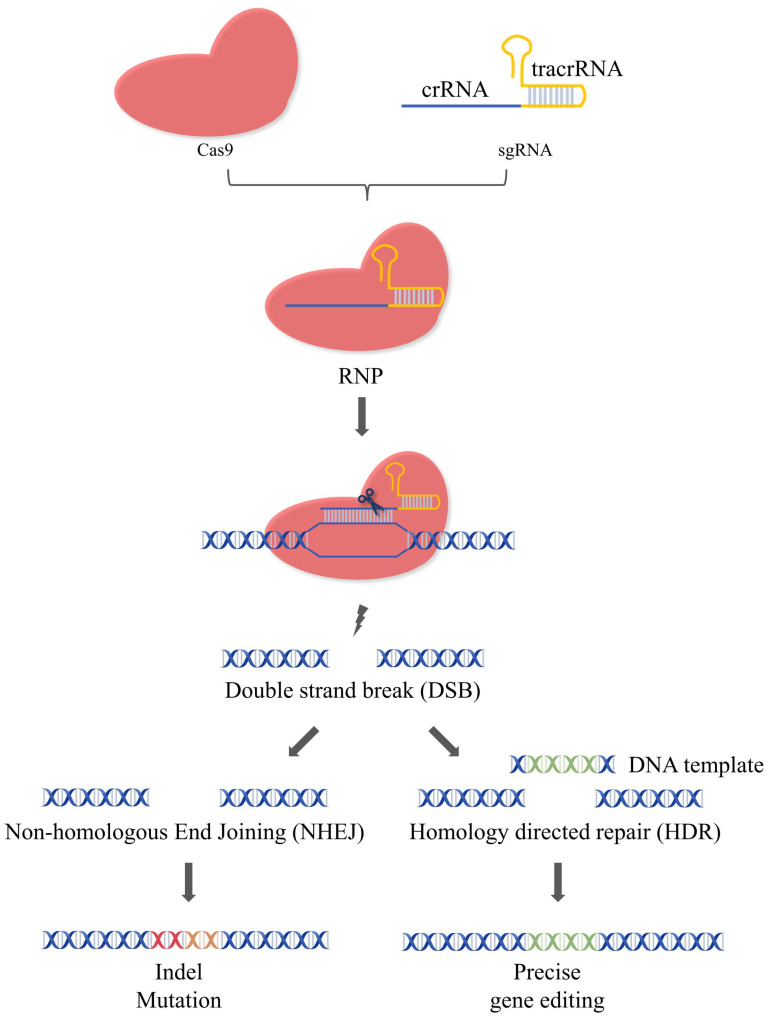
Mechanism of the CRISPR/Cas9 system. The CRISPR/Cas9 system is composed of Cas9 and sgRNA. The Cas9 can specifically cleave the target gene under the guidance of sgRNA to generate DSBs. The cell can repair the DSBs via two pathways: NHEJ or HDR. During NHEJ, indel mutations of edited DNA strands often occur, leading to the frameshifts and/or the formation of premature termination codons. During HDR, the donor DNA template is inserted at the specific sites for precise gene editing.

CRISPR/Cas9 stemmed from the adaptive immune systems of most archaea and many bacteria and has been widely utilized in genome editing [31]. The natural CRISPR-Cas9 system are composed of Cas9, crRNA, and tracrRNA. The crRNA contains a sequence for target DNA recognition and a sequence for tracrRNA binding. The tracrRNA can bind with the tracrRNA via complementary pairing. In mammalian genome editing with CRISPR-Cas9, crRNA, and tracrRNA have been engineered into sgRNA. Therefore, the artificial CRISPR/Cas9 system usually consists of two components: the Cas9 endonuclease and the sgRNA, which form the ribonucleoprotein complex via base pairing to mediate the gene editing. That is, the Cas9 is directed to the upstream of protospacer adjacent motif (PAM) under the guidance of sgRNA and cleaves the target gene to generate the double-strand breaks (DSBs). DSBs can be repaired by cells via two pathways: non-homologous end joining (NHEJ) and homology-directed repair (HDR) [31,32]. The insertion/deletion (InDel) of edited DNA strands often occurs during NHEJ, leading to the frameshifts and/or the premature of stop codons [33]. Unlike NHEJ, which joins the breaks together, the donor DNA template was inserted at the specific sites during HDR to edit the gene accurately (Figure 1). Although both pathways exist in cells simultaneously, only 25% of genome repair occurs via the HDR pathway, while the remaining 75% of DSBs are repaired by the error-prone NHEJ mechanism [34,35]. Strategies to improve the efficiency of HDR have been developed, such as using NHEJ inhibitors or HDR enhancers [36]. 

Different from conventional gene editing tools like ZFNs and TALENs, it is more convenient and easier to personalize the CRISPR/Cas9 complex by only changing the sgRNA sequence [37], rendering it possible to edit multiple independent sites simultaneously. Thus, CRISPR/Cas9 gene editing technology has been broadly utilized to correct mutated genes to treat various diseases, including Duchenne muscular dystrophy (DMD) [38], hereditary tyrosinemia I [39], hypercholesterolemia [40], and cancer [41]. Since the main cause of cancer is the dysregulation of cell growth, knocking out the oncogenes or repairing the tumor-suppressive genes by CRISPR/Cas9 gene engineering tools has shown promising potential in cancer treatment. Up to now, CRISPR/Cas9-based therapy has been utilized to treat multiple tumors, including lung cancer, breast cancer, colon cancer, melanoma, hepatocellular carcinoma, etc. The therapeutic targets for CRISPR/Cas9-mediated cancer treatment include oncogenes (Kras) [7], cell death-related genes (MTH1) [9], epigenetic genes (DNMT1) [42], immune-related genes (CD47) [43], viral oncogenes (E6 or E7) [44], and tumor microenvironment-associated gene targets (VEGFA) [12], etc.

### 2.2. Three Forms of CRISPR/Cas9 Delivery

**Figure 2:**
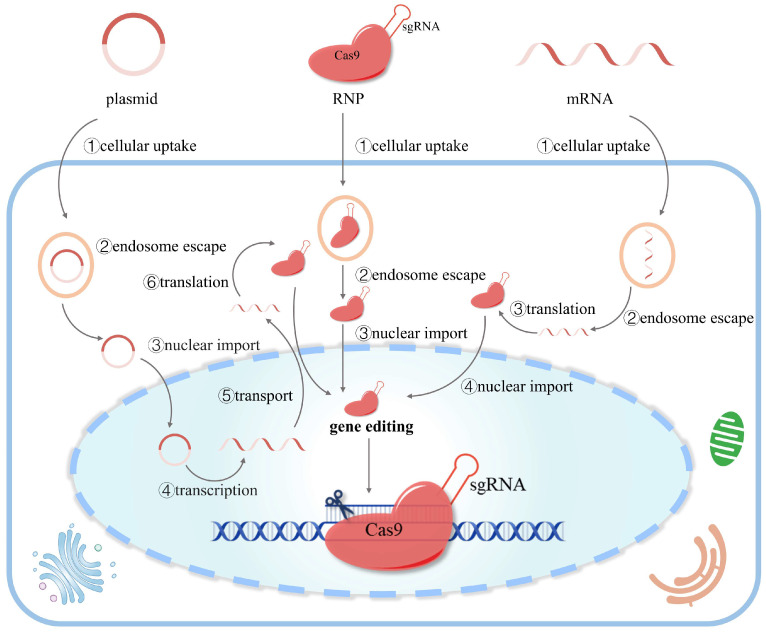
Different forms of CRISPR/Cas9-based gene editing. There are three strategies to edit the genome using CRISPR/Cas9: DNA (plasmid), mRNA, and ribonucleoprotein (RNP). The plasmid has to undergo multiple biological processes (cellular uptake, endosome escape, nuclear import, transcription, transport, and translation) to express the RNP for efficient gene editing. mRNA is translated to RNP in the cytoplasm and then transported to the nucleus for gene editing. RNP can initiate gene editing after entering the nucleus without transcription or translation.

There are three forms of delivery when using the CRISPR/Cas9 system: DNA, mRNA, and ribonucleoprotein (Figure 2). Each of these delivery forms has advantages and disadvantages in efficiency and accuracy. Regardless of the different delivery forms, the ribonucleoprotein complex formed by Cas9 and sgRNA is the key to gene editing.

#### 2.2.1. DNA (Plasmid)-Based Delivery

Cas9 and sgRNA cassettes can be packed together in the same plasmid or separately in two plasmids. After transcription and translation, the formed ribonucleoprotein complex enables gene editing at the target locus. Due to its good stability and relatively low cost, DNA-based gene engineering may be easier to scale up and translate into the clinic. However, the large size of Cas9 and the plasmid makes it even harder for efficient delivery. An additional obstacle for DNA-based gene editing is the requirement for multiple biological processes, including transcription and translation, which delay the onset of gene editing and may decrease the editing efficiency. Moreover, the continuous expression of ribonucleoprotein increases the risk of off-target mutagenesis [19].

#### 2.2.2. mRNA-Based Delivery

Cas9 mRNA can be obtained by in vitro transcription and mediate genome editing after translation in cells. Usually, mRNA-based delivery enables a quicker onset of gene editing than DNA-based delivery, as it does not require nuclear entry for DNA transcription. The transient expression of Cas9 mediated by mRNA also reduces the risk of off-target effects. However, mRNA is less stable than DNA and is prone to degradation, increasing the difficulty of production, storage, and clinical use. In addition, when delivering in the form of mRNA, the right timing of delivery has to be taken into account. For efficient genome editing, Cas9 and sgRNA should be presented at the target site simultaneously. Therefore, delivering the mRNA into the cells before sgRNA is optimal since the mRNA encoding Cas9 has to be translated first. One study showed that injection of Cas9 mRNA followed by an injection of sgRNA 6 h later in mice is helpful to increase the efficiency of gene editing [45].

#### 2.2.3. Protein-Based Delivery

It is the most straightforward way to deliver the ribonucleoprotein complex formed by Cas9 and sgRNA. It does not require these biological processes, such as transcription and translation, thus enabling the quickest onset of gene editing [46]. However, the large size of Cas9 protein (160 kDa) and gRNA (34 kDa) increases the difficulty of delivery. Moreover, it is difficult and costly to obtain proteins of high purity, and proteins isolated from bacteria may contain toxins, increasing the risk of safety. Additionally, the use of proteins in the body may trigger immune responses.

### 2.3. Multiple Barriers of Delivery

High molecular weight and massive charge are the common characteristics of DNA, mRNA, and ribonucleoprotein, and the use of these biomacromolecules all face similar dilemmas in their delivery. Firstly, these cargoes should be packed with suitable materials to facilitate delivery. Then, during circulation, these biomacromolecules should resist the harsh environment in vivo. Especially for mRNA and proteins of poor stability, it is vital to protect them from enzymes to maintain their stability for in vivo application. Also, it is essential to prevent clearance by the mononuclear phagocyte system (MPS) and to maintain long circulation time for efficient genome editing [47]. Moreover, the efficiency and accuracy of CRISPR/Cas9-based gene engineering relies on the accumulation of these cargoes at their target organs, cells, or even organelles. After accumulating at their target organs, they have to cross the extracellular matrix and accurately identify the target cells. However, the CRISPR/Cas9 machinery is unlikely to pass through the cell membranes without the assistance of a carrier due to their high molecular weight and massive charge. In addition to cellular uptake, intracellular barriers, including endosomal escape and nuclear entry, also severely restrict the editing efficacy. For mRNA and proteins whose target sites are in the cytoplasm, the key to delivery is cellular uptake and endosomal escape, while for DNA, whose target sites are in the nucleus, the determining step is nuclear entry. Therefore, it is crucial to design suitable delivery systems on the basis of their own characteristics to maximize the genome editing efficiency.

## 3. CRISPR/Cas9-Based Delivery Strategies

As mentioned above, the effective delivery of the CRISPR/Cas9 system to the target sites is critical to gene editing efficacy. Many strategies have been utilized to improve the delivery efficiency of the CRISPR/Cas9 system, which can be classified as physical methods, viral vectors, and non-viral vectors. The physical approaches, via transient membrane disruption, mainly include electroporation, membrane deformation, sonoporation, lance array nanoinjection (LAN), microinjection, and hydrodynamic injection [48]. For example, electroporation, the most commonly used physical method in delivering CRISPR/Cas9 tools, enhances the permeability of cell membranes via an electric field, thus facilitating the cellular entry of CRISPR/Cas9 machinery [49]. Despite its high efficiency in vitro, its use for CRISPR/Cas9 delivery in vivo is rare. Another approach, hydrodynamic injection, can promote the formation of pores in the cell membranes via hydrodynamic pressure to promote the entry of macromolecules. Although this method has been applied in delivering CRISPR/Cas9 tools, concerns about the high risk of severe damage to the liver still remain [48].

As naturally evolved infectious agents, viral vectors are efficient delivery systems with high transfection efficiency. Currently, the main viruses for CRISPR/Cas9 delivery are lentiviruses, adenoviruses, and adeno-associated viruses (AAVs). One disadvantage of lentiviruses is the potential risk of integration into the host genome. Alternatively, the use of AAVs can avoid the risk of integration into the host cell, but their use is limited by their loading capacity. For example, the size of spCas9 is about 4.1 kb while the size limitation of the payload of AAVs is about 4.7 kb [29] and, thus, spCas9 and sgRNA have to be packed in two viral vectors separately, which may decrease the efficiency of gene editing. With the development of gene editing technology, the emergence of new versions of nucleases can improve the loading efficiency. For example, the variant of Cas9—SaCas9 or Cpf1 can be packaged in the same viral vector with sgRNA due to their smaller size [50]. Moreover, the immunogenicity of viral vectors is another obstacle to their clinical application.

Another option is the use of non-viral vectors for delivering CRISPR/Cas9 tools, and most of them are in the form of nanoparticles. Due to their safety, stability, and biocompatibility, nanoparticles have demonstrated great potential for in vivo therapy [3,29,51]. The use of these nanoparticles can improve the stability of biological macromolecules and prevent them from degradation during circulation. Moreover, the half-life of nanoparticles in the body can be prolonged through pegylation. Various functional modules can be introduced into the nanoparticles to endow the active targeting at the disease site via facile modification. Furthermore, loading CRISPR/Cas9 tools in the nanoparticles can overcome multiple intracellular barriers of CRISPR/Cas9-based delivery by facilitating cellular uptake, endosomal escape, and nuclear targeting. As the properties (size, shape, and zeta potential) of nanoparticles play a critical role in determining their fate in vivo [52,53], it is essential to characterize these nanocarriers by means of dynamic light scattering (DLS), transmission electron microscope (TEM), scanning electron microscope (SEM), etc. DLS is a common technique for measuring submicron particle size, which can quickly measure the mean hydration diameter, size distribution, and zeta potential of particles. The morphology of the particles can be observed with the assistance of TEM and SEM, which provide direct evidence for confirming the formation of nanoparticles.

**Figure 3:**
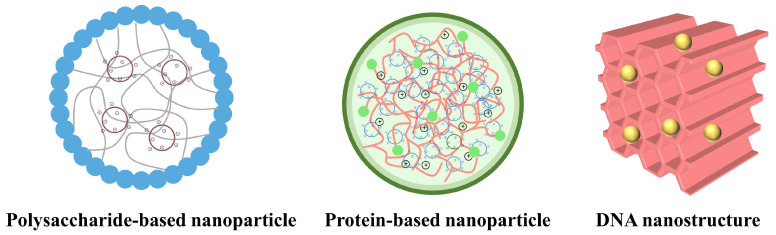
Schematic illustration of natural biopolymer-based delivery systems for CRISPR/Cas9-based gene editing.

Up to now, varieties of nanoparticles have been applied in the CRISPR/Cas9 delivery field, including micelles, dendrimers, liposomes, lipid nanoparticles, metal-organic frameworks (MOFs) [54], and gold nanorods (GNRs) [55], etc. Among these nanomaterials, polymers have recently emerged as appealing materials and have dramatically increased the potential of CRISPR/Cas9 formulations. Polymers are more rigid and stable than liposomes and can be fabricated into various nanostructures with tunable sizes and different surface properties. Another advantage of utilizing polymers is their low toxicity and facile modification. Natural biopolymers are preferred over synthetic polymers for CRISPR/Cas9 delivery due to their biocompatibility, biodegradability, and low immunogenicity. Different from synthetic polymers, natural biopolymers derive from biological sources and are biosynthesized by living organisms such as plants, animals, and microorganisms. Based on their repeating units, natural biopolymers can be categorized into polysaccharides, proteins, and polynucleotides [56]. Numerous natural biopolymers, including chitosan, protamine, and DNA [57,58,59], have been broadly utilized in drug delivery as well as in CRISPR/Cas9 delivery (Figure 3). For example, chitosan, with an inherent positive charge, has emerged as a promising candidate for condensing DNA to enhance transfection efficiency [58]. In the next section, we will summarize the advances of natural biopolymers in CRISPR/Cas9 delivery.

### 3.1. Polysaccharide-Based Delivery of CRISPR/Cas9

#### 3.1.1. Chitosan

Chitosan, a cationic polysaccharide derived from the deacetylation of chitin, has gained great attention due to its biocompatibility and nontoxicity. It consists of repeating units of β-(1–4) N-acetyl glucosamine and D-glucosamine with native amine groups. The physicochemical property of chitosan is determined by molecular weight, degree of deacetylation, conformation, and the pH of the surrounding medium. For example, it is soluble at pH below 6.5 when amino groups are protonated and become positively charged. However, it is insoluble in neutral and alkaline pH conditions due to the hydrophobic effect of the chitosan backbone and the strong intermolecular hydrogen bonding formed by hydroxyl and amino groups, which severely hinders its application in the design of drug delivery carriers [60,61]. Besides, the cytotoxicity of chitosan increases with its molecular weight and degrees of deacetylation, and chitosan with branched structure shows higher cytotoxicity than their counterparts of linear structure. Usually, chitosan with a molecular weight higher than 100 kDa is less biocompatible. Thus, chitosan with a molecular weight ranging between 2.8 and 30 kDa is commonly used in drug and gene delivery due to its biocompatibility [62]. As a cationic polymer, chitosan can form a complex with negatively charged nucleic acids to form positively charged polyplexes, which enhances the interactions with the cellular membranes to facilitate cellular uptake [63]. In addition to DNA condensation, chitosan can promote endosome escape of the polyplexes via the “proton sponge effect” due to the presence of amino groups [64]. The transfection efficiency of chitosan depends on multiple factors, including its degree of deacetylation and the molecular weight of the chitosan, pH of media, ratio of chitosan to gene, and cell type. Some chitosan-based CRISPR/Cas9 delivery systems are developed using its pristine backbone, yet the transfection efficiency of pristine chitosan remains relatively low, and several chitosan derivatives have been explored for enhanced gene transfection including carboxymethylation of chitosan (CMC), O-carboxymethyl-chitosan, arginine-chitosan, and trimethyl chitosan (TMC) [64,65]. Moreover, using ligands to improve the active targeting of chitosan has demonstrated great potential in CRISPR/Cas9-based gene editing. In the next section, all these chitosan derivatives will be discussed in the context of chitosan-based CRISPR/Cas9 delivery systems.

Poly(ethylene glycol) (PEG) is a hydrophilic polymer with favorable properties, and PEGylation has been recognized as an effective way to improve solubility and circulation time in the delivery field. A recent study also revealed that modification of PEG could facilitate the diffusion rate of nanoparticles [66]. The major barrier to pulmonary delivery is the massive mucus lining in the airway, and the application of chitosan for pulmonary gene delivery is limited by its mucoadhesive characteristics. In this study, PEGylated chitosan-based nanocomplexes were prepared for aerosol and mucosal delivery of the CRISPR/Cas9 system. Chitosan of different molecular weights was conjugated with poly(ethylene glycol) monomethyl ether (mPEG) with a high mPEG degree of substitution and then complexed with pSpCas9-2A-GFP via electrostatic interactions. The results show that PEGylated chitosan not only improved the mucus-penetration capability of the nanocomplexes but protected nucleic acids from the stresses of nebulization. In the HEK-293 embryonic cell line, the highest transfection efficiency was 15% at an N/P ratio of 5 at pH 6.5 and 6.8.

Enhanced transfection efficiency is another aim for chitosan modification. For instance, Yoshinaga et al. developed a series of phenylboronic acid (PBA)-functionalized chitosan polymers grafted with low molecular weight branched-polyethyleneimine (CS-PEI) through amide coupling for oral CRISPR delivery [67]. The decoration of low molecular weight PEI can improve the transfection performance of chitosan without increasing its cytotoxicity. The grafting of PBA moieties can improve the potency of chitosan in many ways. Firstly, PBA moieties can stabilize the polyplex to protect the encapsulated genes via hydrophobic interactions. Secondly, the PBA can facilitate the transport of polyplex through the mucus layer via receptor-mediated endocytosis and promote the endosomal escape of polyplex via hydrophobic interactions, which synergistically improves delivery efficiency. Additionally, the PBA can form a reversible ester linkage with a diol compound in a hydrophilic state and responds to cytoplasmic adenosine triphosphate (ATP) to promote plasmid release. Their results demonstrated that decoration of PEI and PBA with optimum ratios improved the performance of chitosan-based polyplexes, with better transfection efficiency than lipofectamine 3000 in the human colorectal line (HCT116). The in vivo study also demonstrated the feasibility of PBA-functionalized CS-PEI for oral CRISPR delivery.

Ligand-modified chitosan has also been fabricated to improve the active targeting of the CRISPR/Cas9-based delivery system. Due to the high expression of the folic acid (FA) receptor, Li et al. designed folic acid (FA) and 2-(diisopropylamino) ethyl methacrylate (DPA) double-grafted trimethyl chitosan (TMC) nanoparticles for the co-delivery of doxorubicin (DOX) and survivin CRISPR/Cas9-expressing plasmid. Ref. [58] They confirmed that modification of FA can enhance the internalization of DOX and plasmid in breast cancer cells via the ligand-receptor interactions in vitro. The combination of chemotherapy and gene therapy exhibited the strongest tumor regression (91.0%) in 4T1 tumor-bearing mice, verifying the synergistic effect of DOX and sgSurvivin pDNA. In addition to grafting ligands onto the chitosan via chemical conjugation, an alternative method is to modify the ligands onto the surface of nanoparticles via electrostatic interactions [68]. For instance, a multifunctional nanoparticle based on chitosan was constructed to knock out the CDK11 gene in targeted tumor cells to regulate cell behaviors, in which negatively charged aptamer AS1411 was incorporated into carboxymethyl chitosan via electrostatic interactions for target delivery [69]. Due to the nucleus targeting capability of AS1411 and the cell-penetrating capacity of KALA peptide, the dual modification of KALA and AS1411 ligands endowed the nanoparticles with enhanced delivery efficiency. This multifunctional delivery system showed the highest cellular uptake efficiency of nearly 100% and dramatically downregulated the expression of CDK11, thus inducing cell apoptosis and inhibiting migration.

The delivery of RNP via the chitosan-based nanoparticle into the nucleus for genome editing has also been reported [70]. Pre-protonated chitosan with a low molecular weight of 1 kDa was firstly coated onto the red fluorescent protein (RFP) and then absorbed the negatively charged Cas9 RNPs and single-strand DNA (ssDNA) donors to self-assemble into nanoparticles. Using the HeLa cell line as a model, the researchers verified that the nanocomplexes efficiently entered the cells and colocalized around the nucleus. Besides, the HDR frequency of nanocomplexes in an engineered blue fluorescent protein (BFP) expressing HEK293 cell line was about 12.5%, comparable to that of the commercial reagent lipofectamine (14.5%).

#### 3.1.2. Alginate

Alginate is a natural polysaccharide typically obtained from marine life, such as seaweed or brown algae [71]. Alginates are linear copolymers composed of (1-4)-linked-β-D-mannuronate and α-L-guluronate residues. They have been extensively investigated and used for many biomedical applications owing to their favorable properties, such as biocompatibility, biodegradability, nontoxicity, and low cost. Various alginate derivatives have been synthesized, including amphiphilic alginate derivatives modified by hydrophobic moieties and alginate derivatives conjugated with cell-adhesive peptides, which demonstrate great potential in delivering bioactive materials [71,72]. The alginate-based delivery systems can be classified into nanoparticles, nanoemulsions, and nanohydrogels according to their preparation methods and characteristics. Alginate hydrogels formed through physical or chemical crosslinking of the polymer chains are widely used in tissue engineering, and their physiochemical properties vary with the molecular weight, crosslinking density, and preparation method. Herein, we will focus on the alginate-based nanostructures for gene delivery, with an emphasis on the nanomaterials for CRISPR/Cas9 delivery.

A dual-targeting CRISPR/Cas9 gene editing nanosystem was fabricated to reverse the malignancy of leukemia cells, in which protamine was complexed with CRISPR/Cas9 plasmid to form the core and then modified with T22-NLS peptide and MUC1-specific aptamer incorporated alginate via electrostatic interactions [73]. In this design, the introduction of alginate into the nanostructure can promote the self-assembly of the nanosystem and simultaneously introduce the cancer-targeting aptamer and peptide. The in vitro study showed that this dual-targeting nanosystem can mediate efficient gene editing for CXCR4 knockout in THP-1 cells, thus inducing cell apoptosis and cell cycle arrest as well as inhibiting cell migration and adhesion.

In another study, oxidized glutathione (GSSG)-crosslinked PEI (GP) was used to condense DNA to form the positively charged polyplexes, and then sodium alginate (SA) with negative charge was absorbed onto the surface via electrostatic interactions to form the tertiary complex [74]. As expected, modification of sodium alginate improved the transfection efficiency of the cationic nanoparticles by approximately 50 times as sodium alginate could promote the cellular uptake and DNA release from the nanocomplex. The co-delivery of p53 and KillerRed gene in this nanoparticle synergistically inhibited tumor cell growth and promoted tumor cell apoptosis by p53-mediated apoptosis and KillerRed-mediated photodynamic therapy.

Although most carriers used for gene delivery are cationic, some noncationic gene vectors have also been utilized. For instance, Guo et al. elaborately designed a noncationic, deformable, and tumor-targeted nanolipogel system (tNLG) for CRISPR genome editing to treat triple-negative breast cancer (TNBC) [75]. The tNLG features a unique deformable core−shell nanostructure. The hydrogel core formed by the alginate confined the diffusion of biomacromolecules due to its polysaccharide network, ensuring the efficient encapsulation of plasmid into the shell. Results showed that this noncationic tNLG could effectively transfect the TNBC cells and significantly suppress the expression of Lcn2 due to their distinctive features. Besides, tNLG mediated a potent CRISPR knockout of Lcn2 in TNBC tumors with an editing efficacy of 81% and significantly suppressed tumor growth.

#### 3.1.3. Hyaluronic Acid

Hyaluronic acid (HA) is a natural liner polymer discovered from bovine eyes in 1934. HA is composed of D-glucuronic acid and N-acetyl-D-glucosamine with a molecular weight ranging from thousands to millions of Daltons [76]. It is hydrophilic due to the presence of hydroxyl groups, carboxyl groups, and acetamido groups. HA has demonstrated great potential in biomedical applications, especially in targeted drug delivery for cancer treatment. Modification of HA is broadly utilized to improve the tumor targeting capacity of the nanocarriers as it can specifically recognize the cluster of differentiation (CD) protein CD44, which is highly expressed in varieties of tumors [43,77]. Besides, the HA can be degraded by hyaluronidase (HAase), and this favorable property has been utilized to promote HAase-responsive drug delivery [78,79].

Li et al. constructed a multifunctional artificial virus (RRPHC) for the targeted delivery of CRISPR/Cas9 plasmid to treat ovarian cancer [9]. This nanocarrier featured a “core-shell” nanostructure, with a core composed of plasmid and a cationic polymer, as well as a versatile multifunctional shell consisting of HA, PEG, and a targeted peptide. The introduction of HA has several advantages: firstly, the negative charge of the HA backbone can reverse the positive charge of RRPHC, improving its stability and minimizing their toxicity. Secondly, the binding of the HA and CD44 receptor can improve tumor targeting and enhance the cellular uptake of the nanocarriers. Thirdly, the degradation of HA by the hyaluronidase can promote the release of cargo for efficient editing. Results showed that RRPHC was 131.3 ± 4.2 nm in size with a negative charge of −21.8 ± 1.8 mV, verifying the charge reversal after HA modification. Besides, RRPHC demonstrated good performance regarding cellular uptake, endosomal escape and transfection efficiency, and induced indel mutations at a frequency of ∼44% in SKOV3 cells. RRPHC effectively disrupted MTH1 and, thus, inducing tumor cell apoptosis in a subcutaneous xenograft tumor model of SKOV3 cells. This study verified the feasibility of utilizing RRPHC for targeted delivery of CRISPR/Cas9 tools. Inspired by the excellent performance of this nanocarrier, researchers of this group have developed a series of HA-functionalized nanosystems [79,80,81,82]. For instance, a programmable unlocking nano-matryoshka was constructed to disrupt programmed cell death ligand 1 (PD-L1) and protein tyrosine phosphatase N2 (PTPN2) to reverse the immunosuppression in tumors. The nano-matryoshka PUN@Cas-PT is composed of an oxidative stress-sensitive core and multienzyme-responsive corona, which can release the CRISPR/Cas9 in tumor sites in response to overexpressed metalloproteases (MMPs), hyaluronidase (HAase), and high endogenous reactive oxygen species (ROS) [83]. In another study, they developed a tumor-specific activated nano-domino-CRISPR for the co-delivery of chlorins e6 (Ce6) and CRISPR/Cas9 plasmid targeting the Bcl-2 gene. Similarly, the decoration of HA augmented the accumulation of nanoparticles in tumors and enhanced their cellular entry [9].

Considering the aforementioned merits of HA, we fabricated a dual-targeted nanocarrier for co-delivering CRISPR/Cas9 plasmid targeting CD47 and plasmid for interleukin- 12 (IL-12) expression to promote the macrophage-mediated phagocytosis [43]. Fluorinated PEI of low molecular weight was utilized to condense the plasmids to form the inner core, while HA and tumor microenvironment sensitive peptide (TMSP) linked by PEG were decorated onto the core via electrostatic interactions. We verified that dual modification of HA and TMSP can dramatically improve cellular uptake and promote endosomal escape, thus effectively transfecting B16F10 cells with a transfection efficiency of nearly 70%. The combination of CD47 knockout and IL-12 production synergistically enhanced macrophage-mediated phagocytosis for tumor immunotherapy.

#### 3.1.4. Cyclodextrin

Cyclodextrin (CD) is a naturally occurring cyclic oligosaccharide composed of α(1→4)-linked glucose units arising from the enzymatic degradation of starch and features a basket-shaped topology [84]. CD contains a hydrophobic cavity, which renders the facile encapsulation of bioactive molecules. Up to date, various CD-derived nanomaterials have been designed to improve the pharmacokinetics of drugs, including to improve the bioavailability of poorly soluble or biodegradable drugs, to prevent undesired effects, and to enhance the permeability of biological membranes [85]. The commercially available CDs can be categorized into α-, β-, and γ-CD based on the number of glucose units in the CD, and among them, β-CD is the most commonly used material in the context of drug delivery. Many β-CD-containing nanocarriers have been reported to deliver CRISPR/Cas9 tools.

Decoration of CDs onto the polymer has been demonstrated to improve the efficiency of gene delivery systems, possibly due to their molecular inclusion, membrane disturbing, and macromolecule shielding capability [86,87,88]. For example, a cationic polymer polyethyleneimine-β-cyclodextrin (PC) was reported to mediate efficient delivery of siRNA and small plasmid as well as large plasmid encoding Cas9 and sgRNA. The genome editing efficiency, determined by Sanger sequencing at two genome loci, hemoglobin subunit beta, and rhomboid 5 homolog 1, was 19.1% and 7.0%, respectively—significantly higher than that of their unmodified counterpart [88]. Similarly, Taharabaru designed a poly(amidoamine) dendrimer (PAMAM)/glucuronylglucosyl-β-cyclodextrin conjugate-based carrier, which showed excellent gene- and siRNA-transferring activities. Its potency in RNP delivery was also verified, which showed higher genome editing activity than PAMAM, lipofectamine 3000, or lipofectamine [89].

**Figure 4:**
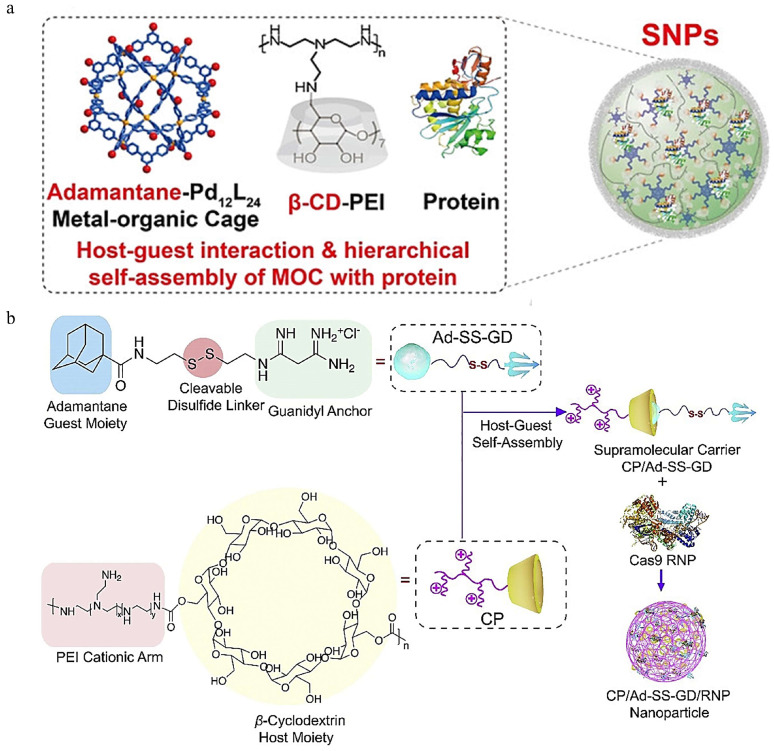
CD-mediated CRISPR/Cas9 delivery. (**a**) Illustration of the self-assembly of adamantane−functionalized M_12_L_24_ MOC and β−cyclodextrin-conjugated polyethyleneimine (PEI−βCD) along with proteins into supramolecular nanoparticles for intracellular protein delivery, reproduced with permission [90] (Copyright 2021, Wiley Online Library). (**b**) Schematic illustration of the preparation of CP/Ad−SS−GD/RNP nanoassembly and intracellular RNP delivery mediated by CP/Ad−SS−GD, reproduced with permission [91] (Copyright 2020, Elsevier).

Hierarchical self-assembly is a powerful strategy to fabricate supramolecular nanostructures for biomacromolecule delivery. Liu and coworkers reported a CD-based polymeric assembly for ribonucleoprotein delivery. The supramolecular nanoparticles were obtained by the assembly of adamantane-functionalized M_12_L_24_ MOC (Ada-MOC) β-cyclodextrin-conjugated polyethyleneimine (PEI-βCD) via the host–guest interaction of Ada-MOC and PEI-βCD, during which the protein was encapsulated [90] (Figure 4a). Using several functional proteins (GFP, RNase A-NBC, and Cas9/sgGFP) as models, they found that this supramolecular nanostructure could maintain the activity of the loaded protein. The gene editing efficiency of the Ada-MOC/PEI-βCD nanoparticle was estimated to be 40% in GFP-HEK293 cells, demonstrating the potential of the nanoparticle for intracellular protein delivery and CRISPR/Cas9 genome editing.

In another study, Wan et al. developed a supramolecular polymer system for delivering Cas9 RNP targeting the mutant KRAS gene [91] (Figure 4b). The disulfide-bridged biguanidyl adamantine (Ad−SS−GD) and β−cyclodextrin-conjugated low-molecular-weight polyethyleneimine (CP) can form stable nanocomplex through supramolecular assembly due to the host–guest interaction between Ad−SS−GD and CP. Cas9 RNP was loaded onto the nanocomplex via multiple strong hydrogen bonds and salt bridges. The inclusion of disulfide bonds endows the nanocomplex with redox-responsiveness, and Cas9 RNP can be released in the reductive intracellular microenvironment. Both Sanger sequencing results and the T7E1 assay verified the excellent genomic disruption mediated by CP/Ad−SS−GD/RNP in vitro. Moreover, targeted KRAS disruption mediated by HA-decorated CP/Ad−SS−GD/RNP nanocomplex was demonstrated to inhibit tumor growth and metastasis in colorectal cancer models. Likewise, a versatile polyplex capable of delivering plasmid, messenger RNA, and Cas9 RNP, was constructed utilizing the host–guest interaction between Ad and β−CP [92].

### 3.2. Protein-Based Delivery of CRISPR/Cas9

Protamine is a native protein obtained from fish and is composed of a group of heterogeneous polycationic peptides, with nearly 67% of its amino acid composition being arginine [93]. Due to its good water solubility, excellent biocompatibility, and unique pharmacological activity, protamine has common uses in clinical practice, such as reversing the anticoagulant function of heparin and prolonging the adsorption of insulin. Usually, protamine from fish has an average molecular weight of about 4500 Da. It can bind to DNA and form nucleoprotamine; this merit has been widely used to condense DNA for gene delivery [94,95]. However, the nanocomplex formed by protamine and DNA is unstable due to insufficient molecular weight and charge density of protamine. Therefore, other liposomal or polymeric vectors are utilized in combination with protamine to improve their stability and transfection efficiency [96,97].

**Figure 5:**
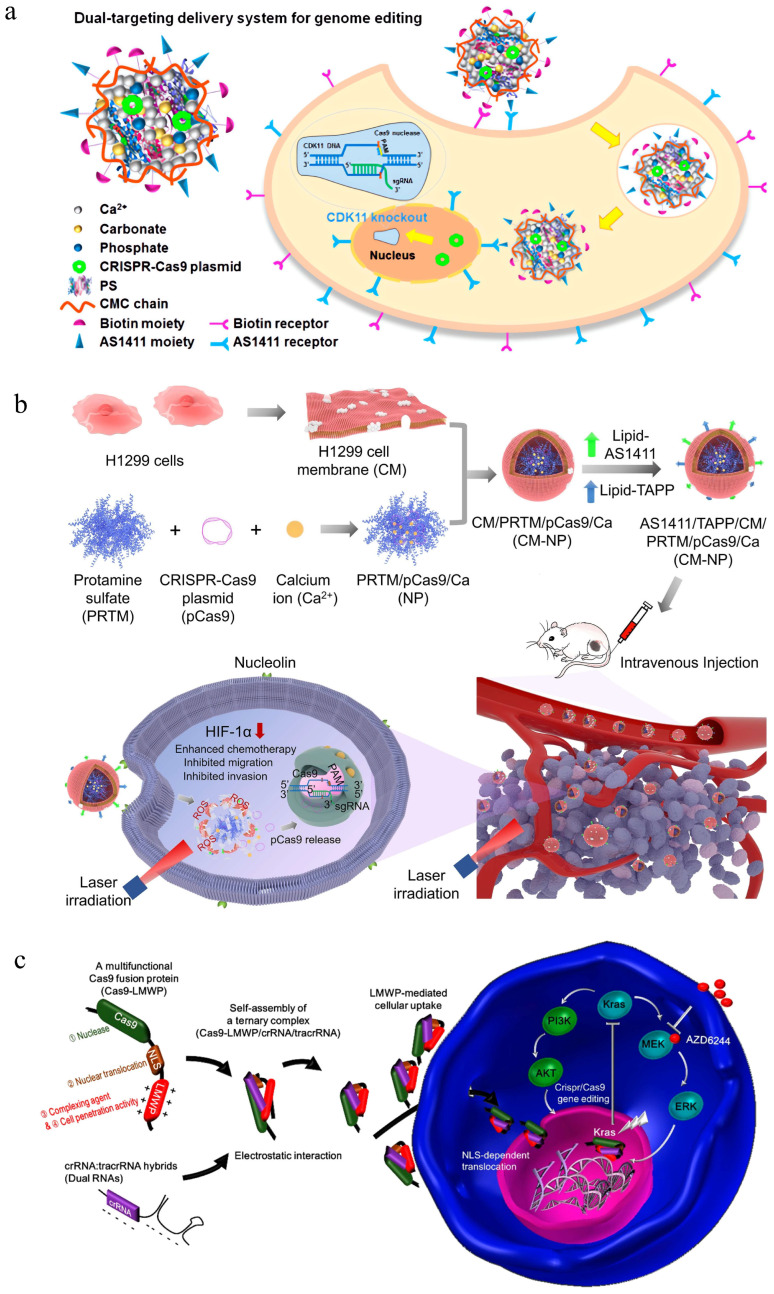
Protamine-mediated CRISPR/Cas9 delivery. (**a**) The dual-targeting delivery system for tumor targeting delivery of CRISPR/Cas9 plasmid to realize effective genome editing, reproduced with permission [98] (Copyright 2018, American Chemical Society). (**b**) Preparation of the engineered biomimetic gene editing system for delivery of the CRISPR/Cas9 plasmid to the targeted tumor cell for gene editing, reproduced with permission [100] (Copyright 2023, Elsevier). (**c**) Scheme of a ternary Cas9 RNP-mediated gene editing for cancer therapeutics, reproduced with permission [101] (Copyright 2018, American Chemical Society).

Cheng and her group have reported a series of protamine-based delivery systems for gene therapy and genome editing. Components, including calcium carbonate, calcium phosphate, and chitosan, have been introduced to improve the transfection performance of protamine-based vectors [57,94]. For instance, the CRISPR/Cas9 plasmid targeting CDK11 was loaded in the core composed of protamine sulfate, calcium carbonate, and calcium phosphate by coprecipitation, and targeted segments composed of biotinylated carboxymethyl chitosan and AS1411 ligand-incorporated carboxymethyl chitosan were decorated onto the surface of a nanovector to form the targeted gene editing system [98] (Figure 5a). This nanovector was proved to efficiently deliver the plasmid to the cell nuclei to mediate genome editing and decrease the CDK11 protein by 70%. Its excellent transfection performance contributed to the dual-targeting ability mediated by the biotin ligands and AS1411 ligands as well as the nuclear targeting capability mediated by protamine.

An alternative method to improve the delivery efficiency of protamine-based carriers is the inclusion of lipids. Zhang et al. constructed a polyethylene glycol phospholipid-modified cationic lipid nanoparticle with a core composed of protamine, plasmid, and chondroitin sulfate, as well as a shell containing cationic lipids (DOTAP). As expected, the decoration of cationic lipids protected nucleic acids from degradation and promoted efficient endosomal escape, resulting in 47.4% transfection efficiency in A375 cells in vitro. The intratumor injection of this nanoparticle significantly disrupted pololike kinase 1 and suppressed tumor growth in vivo [99].

Recently, a cell membrane biomimetic core–shell system for light-controllable precise gene editing has been reported [100]. In this core–shell system, the inner core was formed by protamine, CRISPR/Cas9 plasmid targeting HIF-1α and calcium ions via electrostatic interactions, which was further decorated with a cell membrane modified by AS1411 aptamer and a photosensitizer (Figure 5b). This nanosystem enabled efficient genome editing both in vitro and in vivo due to the enhanced permeability mediated by Ca^2+^, the improved stability and tumor targeting mediated by the cell membrane, as well as the light-controllable endosome disrupting ability mediated by the photosensitizer. In the H1299-xenograft model, genetic knockout of HIF-1α enhanced the therapeutic efficacy of paclitaxel, thus inhibiting tumor growth and metastasis.

In addition to plasmid delivery, protamine-based nanomaterials have also demonstrated great potential in RNP delivery. Kim et al. developed a multifunctional Cas9 fusion protein (Cas9-LMWP) for self-delivery of Cas9 RNP [101]. The Cas9 fusion protein contained a low molecular weight protamine (LMWP) and a nuclear localization sequence, which can direct the self-assembly of Cas9 and RNA hybrids (Figure 5c). This ternary Cas9 RNP enabled efficient delivery to the cells owing to its nuclear translocation ability. Both in vitro and in vivo studies demonstrated the KRAS-disrupting ability of this nanoparticle in lung cancer.

### 3.3. Polynucleotide-Based Delivery of CRISPR/Cas9

Double-stranded DNA is a naturally occurring nanoscale material with a diameter of about 2 nm and a length of about 3.4 nm per helical turn. In the past decade, DNA nanotechnology has demonstrated great potential in many fields, such as bioimaging, biosensing, and drug delivery [102]. A series of DNA-based nanostructures have been developed for the therapeutic oligonucleotide delivery ranging from aptamers, small interfering RNA (siRNA) or antisense RNA, and sgRNA, owing to their excellent biocompatibility and biodegradability, high capacity for payloads, and great stability [59,102]. Another advantage of DNA-based nanostructures is that their size and shape can be precisely controlled as the DNA sequences are programmable and the interactions between DNA are predictable, and various methods like tile-based, origami-based, nanoparticle-templated and RCA-assisted methods have been utilized to direct the self-assembly of DNA nanostructures [102]. Some DNA nanostructures, including DNA nanoclew, DNA nanoflower, and DNA nanoframework have been reported to facilitate the delivery of CRISPR/Cas9.

**Figure 6:**
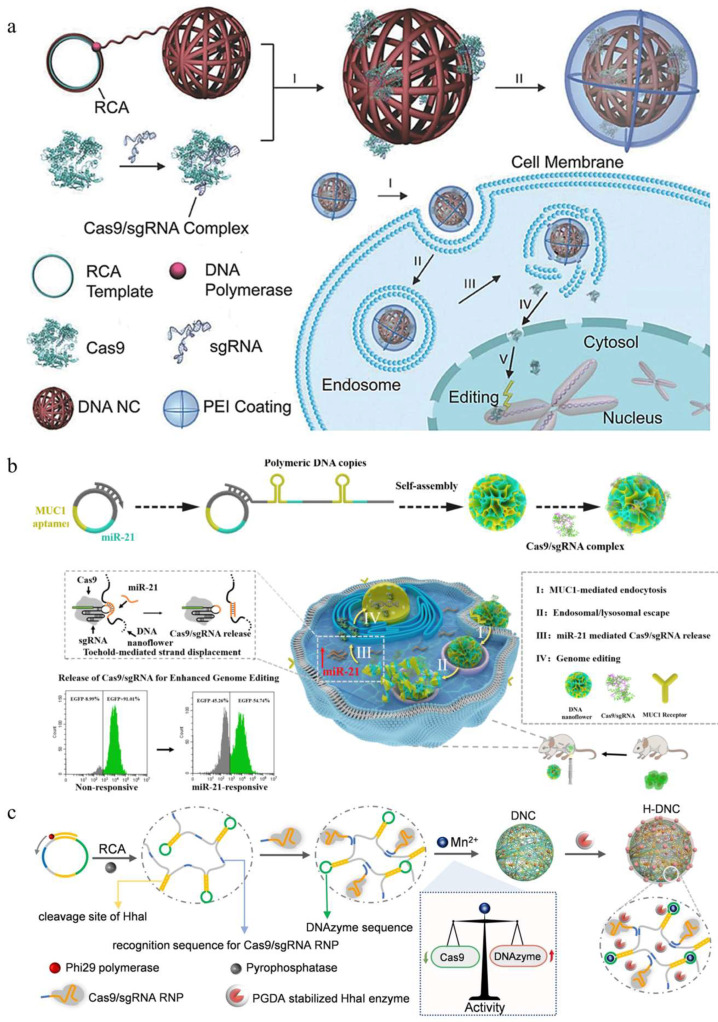
DNA-mediated CRISPR/Cas9 delivery. (**a**) Design of the DNA NC-based CRISPR/Cas9 delivery system, reproduced with permission [103] (Copyright 2015, Wiley Online Library). (**b**) Schematic diagram of microRNA-responsive DNA nanoflower (DNF) for release of Cas9/sgRNA and enhanced genome editing, reproduced with permission [104] (Copyright 2020, Elsevier). (**c**) The molecular design and preparation of H-DNC for the co-delivery of Cas9/sgRNA RNP and DNAzyme, reproduced with permission [105] (Copyright 2022, Wiley Online Library).

Inspired by the ability of single-stranded DNA to base-pair with the guide portion of the Cas9-bound sgRNA, Sun et al. constructed a DNA nanoclew (DNA NC) via rolling circle amplification (RCA) for simultaneously delivering Cas9 and sgRNA [103]. In their design, the Cas9/sgRNA complexes were loaded in the yarn-like DNA NC, and then, PEI was coated onto the surface to facilitate the endosomal escape (Figure 6a). The nanocomplex had a hydrodynamic diameter of approximately 56 nm, and its zeta potential was reversed to +18.6 ± 4.1 mV due to the decoration of the cationic polymer PEI. They evaluated the gene editing efficiency of the DNA NCs in an established U2OS cell line that constitutively expresses a destabilized form of EGFP (U2OS.EGFP), which showed that the PEI-coated DNA NC-induced mutation frequencies of 28%, significantly higher than that of their uncoated counterpart (5%). Intratumoral injection of this nanocomplex led to significant downregulation of EGFP in U2OS. EGFP tumor-bearing mice, further demonstrating its potency for genome editing in vivo.

Inspired by the potential of DNA NCs in Cas9 RNP delivery, the DNA nanoflowers that released RNP in a microRNA-responsive manner were fabricated for cell-specific genome editing [104]. DNA nanoflowers (DNF) were prepared by RCA, which contained multiple replicates of MUC1 aptamers and miR-21 binding sequences, and then RNP was loaded onto the DNFs via sequence hybridization (Figure 6b). Due to the overexpression of miR21 in tumor cells, the Cas9/sgRNA complex was released from DNFs by toehold-mediated sequence displacement, which was verified by fluorescence resonance energy transfer. Moreover, the gene editing efficiency of the DNFs was approximately 21% in HeLa.EGFP cells, about two-fold of that mediated by their non-responsive counterpart. A similar trend of editing efficiency was observed in HeLa.EGFP tumor-bearing mice.

Recently, a proton-activatable DNA-based nanosystem was reported to co-deliver Cas9/sgRNA and DNAzyme for breast cancer therapy [105]. The scaffold of the nanosystem was an ultra-long single-stranded DNA generated via RCA, during which the repeated sgRNA recognition sequences, DNAzyme, and HhaI cleavage sites were programmed in the DNA chain. Then, the Cas9/sgRNA bound to the sgRNA recognition sequences via complementary base pairing. Finally, the acid-degradable polymer-coated HhaI enzyme was loaded to form the proton-activatable DNA-based nanosystem (Figure 6c). Scanning electron microscope (SEM) showed that the DNA-based nanoparticles were about 50 nm in diameter. The DNA release studies showed that the released DNA reached 40% in the medium with pH 5.4 within 4 h, while only 5% DNA was released in the medium with pH 7.4, verifying the proton-responsive DNA release of the nanosystem. The gene editing efficiency of the nanosystem was 40% in vitro. Owing to its efficient delivery, the proton-activatable nanosystem demonstrated a combined antitumor effect of PLK1 disruption by Cas9/sgRNA RNP and EGR-1 silencing by DNAzyme in vivo. Apart from the previously mentioned DNA-based nanostructures for microRNA or proton-responsive drug release, DNA nanoframework for glutathione-responsive disassembly has also been synthesized to control the delivery of CRISPR/Cas9 system [59], highlighting the potential of stimuli-responsive DNA nanosystems in genome editing.

## 4. Conclusions and Outlooks

CRISPR/Cas9 has been engineered as an efficient and versatile gene editing tool and is extensively used to treat a variety of diseases, especially cancer. CRISPR/Cas9 targets multiple genes for cancer treatment, including oncogenes, cell death-related genes, epigenetic genes, immune-related genes, viral oncogenes, and tumor microenvironment-associated gene targets. However, the broad application of CRISPR/Cas9 techniques in biomedicine is confined by the delivery efficiency and immunogenicity of the vectors. For example, viral vectors, one of the most popular CRISPR/Cas9 delivery strategies, are associated with the risk of genotoxicity and immunogenicity despite their relatively high transfection efficiency. Besides, limited packaging capacity and the high cost of production limits their clinical translation. Alternatively, non-viral vectors have grown exponentially and revolutionized the field of gene editing. A series of nanoparticles have been utilized for CRISPR/Cas9 delivery, including micelles, dendrimers, liposomes, SLNs, MOFs, MSNs, and AuNPs. Compared to viral vectors, nanoparticles possess favorable properties, such as low toxicity, ease of production and facile modification. Among these nanoparticles for CRISPR/Cas9 delivery, natural biopolymer-based carriers are particularly promising candidates due to their low immunogenicity, intrinsic biological activities, great stability, as well as excellent biocompatibility and biodegradability. These biopolymers can be further modified to improve their solubility and stability, DNA condensing ability, delivery efficiency, as well as stimuli-responsiveness. Natural biopolymer-based delivery systems that have been exploited for CRISPR/Cas9 delivery are summarized in Table 1.

The rapid development of natural biopolymer-based gene editing systems, particularly for the treatment of cancer, is reassuring, but several challenges remain in this field for their clinical translation. Firstly, the transfection efficiency of natural biopolymers is inferior to that of viral vectors or synthetic polymers. Therefore, in some cases, synthetic polymers such as low molecular weight PEI or PAMAM were introduced, either by electrostatic adsorption or chemical conjugation, into the natural biopolymer-based delivery system to improve the delivery efficiency. However, the non-biodegradability of these synthetic polymers in vivo may increase the toxicity in the long term. Secondly, utilizing the intrinsic receptor-binding ability of natural biopolymers (e.g., HA) or decoration of tumor-targeting ligands onto the natural biopolymer-based carriers has been widely used to improve the tumor-targeting ability of the delivery system. However, the accumulation of nanoparticles in other organs (e.g., liver, lung, spleen, kidney) was also observed, which may increase the risk of off-target genome editing in other tissues and organs. Thirdly, the inclusion of functional modules (e.g., aptamer, disulfide bond, TAT peptide) indeed improves the delivery efficiency of nanocarriers to some extent by enhancing cellular uptake, promoting endosomal escape, accelerating cargo release as well as facilitating nuclear entry, which also complicates the carrier design, making it difficult for large scale production. Moreover, as natural biopolymers derive from biological sources and are biosynthesized by living organisms such as plants, animals, and microorganisms, natural biopolymers of low purity may contain a minority of microbes. Therefore, proper techniques should be used to obtain natural biopolymers of high purity, and it is important to measure microbial contamination to minimize the risk of microbial contamination. Furthermore, it should be noted that the gene editing efficiency and safety in vivo are determined by both the CRISPR/Cas system and the nanocarrier. Therefore, when designing a delivery system for gene editing, one should choose the proper delivery modes of the CRISPR/Cas9 system (DNA, mRNA, and ribonucleoprotein) based on their own characteristics. It is also crucial to optimize the CRISPR/Cas system to decrease the potential risk of off-target events, such as screening the sequence of sgRNA and using some more specific nucleases (e.g., eSpCas9, SpCas9-HF1 [106,107]). We believe that further advances in nanotechnology and gene editing techniques will open infinite possibilities for tumor therapy.

## Figures and Tables

**Table 1:** Summary of natural biopolymer-based delivery systems.

Types	Advantages and Disadvantages	CRISPR/Cas9 Systems	Targets	Cells	Refs.
**Chitosan**	Good adsorptive property Facile modification Poor solubility Low transfection efficiency	Plasmid	Survivin	4T1 cells	[58]
		Plasmid	PCSK9 and ANGPTL3	HEK293T and HCT116 cells	[67]
		Plasmid	FOXM1	MCF-7, HeLa, HEK293 and SK-MES-1 cells	[68]
		Plasmid	CDK11	MCF-7 cells	[69]
		RNP	PRDX4	HEK293T, RAW264.7, HeLa, U2OS and A549 cells	[70]
**Alginate**	Stability Hydrogel formation in water Mucoadhesive and mucopenetrating FDA approved Low encapsulation efficiency	Plasmid	CXCR4	THP-1 cells	[73]
		Plasmid	Lcn2	TNBC cells	[75]
	High affinity to CD44 receptor Degraded by hyaluronidase Easy to functionalize Short lifetime	Plasmid	MTH1	SKOV3 cells	[9]
		Plasmid	CD47	B16F10 cells	[43]
		Plasmid	Bcl-2	B16F10 cells	[79]
		Plasmid	PD-L1 and PTPN2	B16F10 cells	[83]
**Cyclodextrin**	Facile modification Facile encapsulation of bioactive molecules Improve the stability of encapsulated cargoes Modification required for DNA condensation	Plasmid	HBB and RHBDF1	HeLa cells	[88]
		RNP	AAVS1	SH-SY5Y cells	[89]
		RNP	GFP	GFP-HEK293 cells	[90]
		RNP	KRAS	SW-480 cells	[91]
		RNP	BFP	BFP-expressing HEK293 cells	[92]
**Protamine**	DNA condensation ability FDA approved Inherent pharmacological activity Prone to aggregation in circulation	Plasmid	CTNNB1	HeLa cells	[57]
		Plasmid	FAK	HeLa cells	[94]
		Plasmid	CDK11	HEK293T and MCF-7 cells	[98]
		Plasmid	PLK1	A375, PC3, and MCF-7 cells	[99]
		Plasmid	HIF-1α	H1299 cells	[100]
		RNP	KRAS	A549 cells	[101]
**DNA**	Great stability Can be engineered with a defined structure High capacity for payloads Modification required to improve delivery efficiency	RNP	PLK1	MCF-7 and BEAS-2B cells	[59]
		RNP	EGFP	U2OS.EGFP cells	[103]
		RNP	EGFP	HeLa.EGFP cells	[104]
		RNP	PLK1	MCF-7 cells	[105]

## Data Availability

Not applicable.
